# Risk prediction of covid-19 related death and hospital admission in adults after covid-19 vaccination: national prospective cohort study

**DOI:** 10.1136/bmj.n2300

**Published:** 2021-09-20

In this paper by Hippisley-Cox and colleagues (*BMJ* 2021;374:n2244, doi:10.1136/bmj.n2244, published 17 September 2021), coauthor Jonathan Valabhji should be linked to affiliation 11 (NHS England and Improvement, London, UK). The article will be updated in due course. 

